# Supplementary figures and images for: RFPDR: a random forest approach for plant disease resistance protein prediction
Source: PeerJ. 2022 Apr 22;10:e11683. doi: 10.7717/peerj.11683 (PMC9037127; doi:10.7717/peerj.11683)

**A**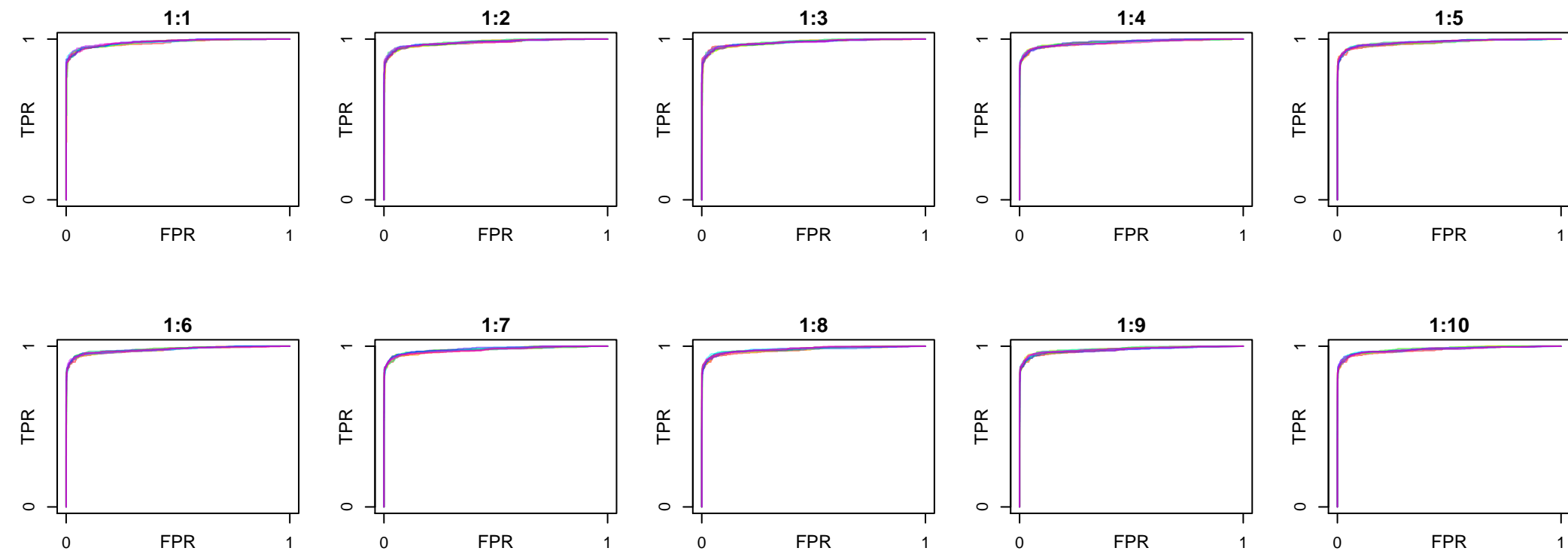**B**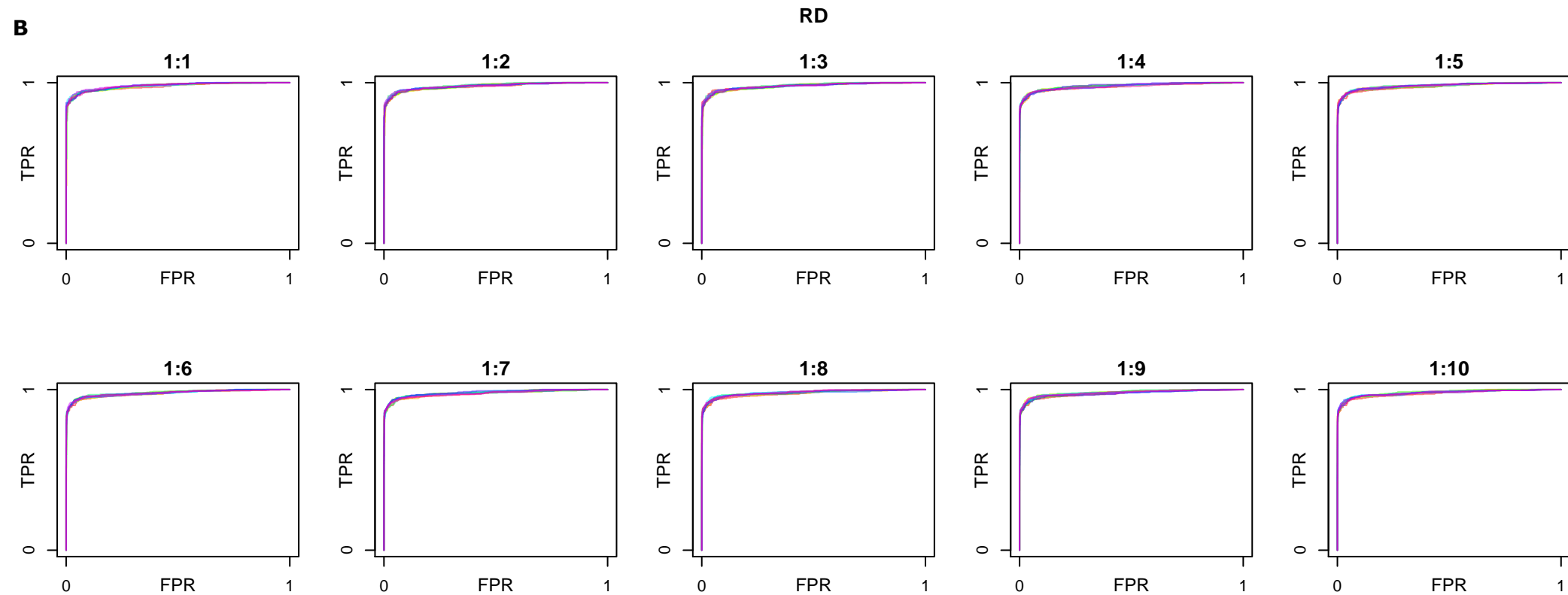

Supplement: Supplemental Information 1 — (A) Full-dimension random-forest plant disease resistance model (FD-RFPDR); (B) Reduced-dimension RFPDR (RD-RFPDR). TPR: true positive rate; FPR: false positive rate. [file peerj-10-11683-s001.pdf]
